# Data-Driven Sustainable *In Vitro* Campaigns to Decipher Invasive Breast Cancer Features

**DOI:** 10.1021/acsbiomaterials.5c00731

**Published:** 2025-07-25

**Authors:** Lekha Shah, Valentina Breschi, Annalisa Tirella

**Affiliations:** † Department of Industrial Engineering and BIOtech Research Center, 19034 University of Trento, 38123 Trento, Italy; ‡ Electrical Engineering Department, 3169 Eindhoven University of Technology, De Zaale, 5600 MB Eindhoven, The Netherlands

**Keywords:** in vitro models, hydrogels, microphysiological systems, breast cancer invasion, machine learning, unsupervised *k*-means, feature importance

## Abstract

The intrinsic complexity of biological processes often hides the role of dynamic microenvironmental cues in the development of pathological states. Microphysiological systems (MPSs) are emerging technological platforms that model *in vitro* dynamics of tissue-specific microenvironments, enabling a holistic understanding of pathophysiology. In our previous works, we engineered and used breast tumor MPS differing in matrix stiffness, pH, and fluid flow mimicking normal and tumor breast tissue. High-dimensional data using two distinctive human breast cell lines (i.e., MDA-MB-231, MCF-7), investigating cell proliferation, epithelial-to-mesenchymal transition (EMT), and breast cancer stem cell markers (B-CSC), were obtained from breast-specific microenvironments. Recognizing that the widespread adoption of MPS requires tailoring its complexity to application demands, we herein report an innovative machine-learning (ML)-based approach to analyze MPS data. This approach uses unsupervised *k*-means clustering and feature extraction to inform on key markers and specific microenvironments that distinguish invasive from non-invasive breast cell phenotypes. This data-driven approach streamlines future experimental design and emphasizes the translational potential of integrating MPS-derived insights with ML to refine prognostic tools and personalize therapeutic strategies.

## Graphical abstract

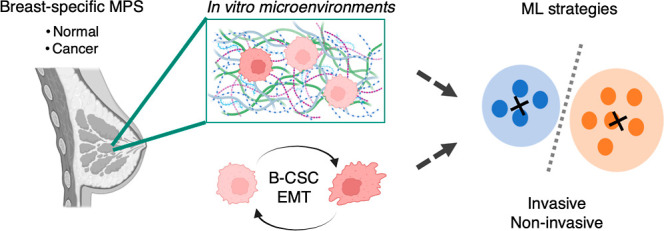

## 1 Introduction

Breast cancer is a significant cause of cancer-related deaths among women, with recent statistics stating 2.3 million cases diagnosed globally in 2022 and an estimated 660,000 deaths.^1^ Most of these deaths are related either to metastatic disease at initial presentation (Stage III and above) or to recurrence at a later stage.^2^ In particular, recurrence occurs in around 20% of breast cancer cases, with the time to recurrence varying from as low as 3 months to as long as 32 years after the initial diagnosis.^3,4^ In this scenario, health technologies are urged to tackle issues such as prevention, precision diagnostics, personalized medicine, and disease management.

In the specific case of breast cancer, improved information on a patient’s risk of recurrence and estimated time to recurrence become very important to guide appropriate and personalized adjuvant therapy, with the possibility to effectively plan future screening programs. In fact, the risk of breast cancer recurrence and metastasis has already been linked clinically to different factors such as molecular subtypes, grade, tumor node status, size of initial tumor,^3^ and patient’s status (age and menopause).^5^ In line with these results, improvements toward a more patient-centered approach in prognostic tests for different stages and identification of appropriate treatment have been implemented.^6,7^ For example, among the molecular subtypes, triple negative breast cancer (TNBC) and Her2+ subtypes have more recurrence risk and lower average recurrence time (26 months) as compared to luminal A and luminal B subtypes (56 months).^3^ Other tools, such as the Nottingham prognostic index, take into consideration only histological factors,^8^ whereas advanced ones such as MammaPrint, Oncotype DX, Mammostrat, Prosigna, EndoPredict, and IHC4 are more patient specific with relevant gene expression and biomarker analysis.^6^

However, all these tests represent a snapshot of each patient’s condition and lack information on the time variance of tumor progression, metastatic onsets, and possible metastatic dissemination. It is known that at the time of diagnosis of breast cancer, an estimated 75% of initial tumors are already disseminated to a distant site.^9^ These micrometastatic sites persist in a state of dormancy within the body until the onset of full metastatic expansion eventually occurs, which is known to correlate with tumor-cell phenotypes and tumor microenvironment (TME). Therefore, identifying which features of the breast tumor ecosystem trigger onset of tumor progression and dissemination is a key enabler for precise prognosis and identification of efficacious treatments.

Preclinical animal models, like patient-derived xenografts, are considered the most promising *in vivo* model to study the spatial structure, heterogeneity, and genomic features of human cancers for identification of effective treatments.^10^ However, these approaches are prone to ethical concerns while also being time-consuming and costly in developing patient-specific and patient-centered models.^10,11^ In addition, such *in vivo* models often do not replicate the rate of dissemination and metastasis due to a poor representation of human-specific metastatic sites.

Preclinical *in vitro* models, often considered too simplistic, can now integrate technological advancements, such as advanced biomaterials in microfluidic technologies and together with digital data and tools are considered key enablers for a more precise and sustainable patient-centered solution to tackle human diseases. The main advantage of engineered *in vitro* models is their ability to mimic multiple aspects of tissue-specific dynamics while precisely controlling the ecosystem, spanning from the selection of cell types to the physical properties of the extracellular matrix (ECM). Thanks to these technological advancements and integrations, engineering tissue-specific key features in in vitro models raises debate over whether these or *in vivo* models serve as better models for understanding tumor dynamics and metastatic onsets.^12,13^

Microphysiological systems (MPSs) are microscale three-dimensional (3D) *in vitro* cell culture platforms modeling the functional features of tissues by exposing cells to a combination of physical (e.g., temperature, pH, oxygen), biochemical, electrical, mechanical (e.g., flow, stretch), structural, and/or morphological conditions to replicate healthy or diseased tissue/organ functions.^14^ In this perspective, we engineered 3D *in vitro* breast cancer models for mechanistic discovery and tailoring TMEs with reproducible and customizable physicochemical properties to match the breast-specific microenvironment.^15^ Such breast-specific MPS enabled independent modulation of physicochemical properties of the ECM (i.e., mechanical properties, density, and composition), environmental pH, and fluid flow (Figure 1). Alginate-based hydrogels were used to tailor compressive moduli in the range of 2–10 kPa, as reported to be clinically relevant in the context of breast cancer matrix stiffness.^16,17^ The combination of mechanically tunable alginate hydrogels with ECM-derived materials (i.e., gelatin) returned interpenetrated hydrogel networks with controlled density, directly linked to clinically relevant markers in breast cancer diagnosis that can be easily integrated in a fluidic chamber to study cell–matrix interaction under fluid flow-mediated force transmission. Based on our experience in analyzing both individual and combinatorial effects of breast-specific TMEs on two selected human breast cancer cell lines (i.e., the highly invasive/recurrent TNBC cell line MDA-MB-231, the less invasive/low recurrence luminal A cell line MCF-7) by measuring their proliferation, breast cancer stem cell (B-CSC) population, and epithelial-to-mesenchymal transition (EMT),^18,19^ we correlated that TME key features can modulate malignant phenotypic transitions such as EMT/MET and also have a far-reaching impact by increasing invasive potential and bone metastatic osteolysis in vitro.^15,20^ This confirms that breast-specific MPS could inform not only on the current molecular state of the tumor but also on its potential to change into malignant phenotypes, given the relevant microenvironmental conditions.

**Figure 1:**
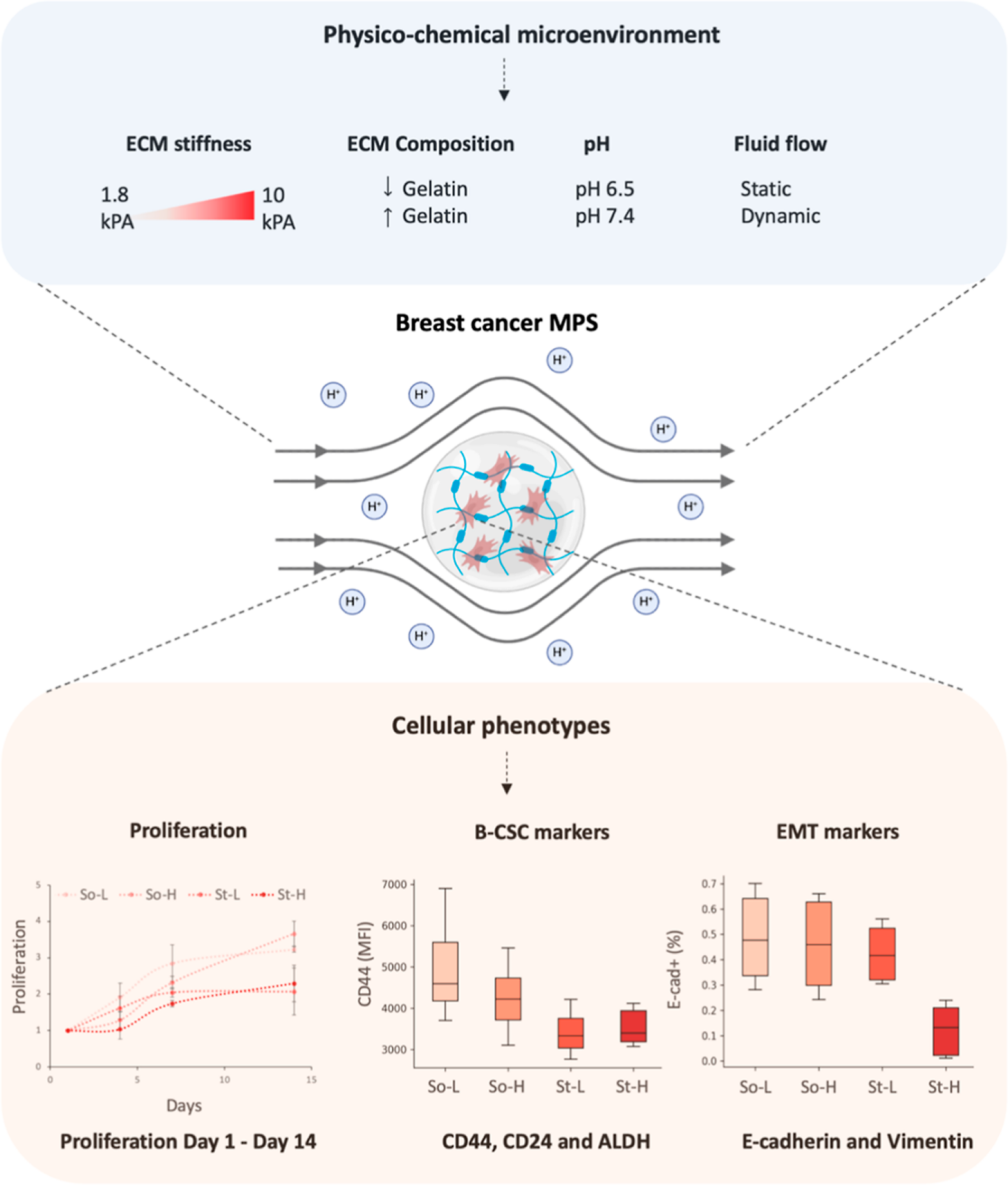
Experimental design representing breast-specific MPS and biological readouts. Schematic representation of human breast cancer cells (i.e., MDA-MB-231, MCF-7) encapsulated in hydrogels with tailored physical properties (i.e., Young’s modulus, composition, density) cultured in controlled culture system (i.e., pH, perfusion). Cellular phenotypes, such as proliferation (Alamar blue assay), flow cytometry analysis of B-CSC marker expression (CD44, CD24, ALDH), and EMT marker expression (E-cadherin and Vimentin), were monitored and recorded over time. Example of obtained data (bottom), cell proliferation curves, CD44 median fluorescence intensity, and E-cadherin+ (%) data of MCF-7 cells cultured in four different hydrogels (So-L, So-H, St-L, and St-H) at pH 7.4 and static conditions.

While designed to offer a better understanding of cell–matrix interactions and linked biological processes, MPS models often require a rather long execution phase to first validate physicochemical features of the TME, followed by time-consuming experimental campaigns with time points decided based on previous studies and not tailored to follow the dynamic of the specific model. Experimental campaigns often collect high dimensional data sets, which increase proportionally to the number of controlled variables of the MPSs used, often resulting in readouts that could be difficult to fully interpret and understand. Indeed, this complexity hinders a clear understanding of each variable’s role, for example, in guiding cell phenotype behaviors, ultimately preventing the identification of parameters of interest to distinguish cellular phenotypes and, therefore, to inform on disease progression (e.g., invasion, distal metastasis). Techniques drawn from statistics and machine learning (ML) can be used to disentangle retrieved information, setting the ground for sustainable design approaches that emphasize relevant TME features influencing the outcome of trials and identify a limited pool of key defining variables. As a consequence, ML not only provides a set of approaches to better understand patterns in cells’ behaviors linked to the TME features, but it can empower future *in vitro* experiment design.

Based on these observations, we herein collected all experimental data sets from previous studies and used a ML approach to better understand the impact of the different engineered features of the breast cancer in vitro models and the relevance of the measured outcomes in shaping and describing specific cellular and biological readouts, with the final goal to achieve a sustainable-by-design experimental campaign and simplify the analysis of trials’ readouts toward a patient-centered predictive model (Figure 1). However, unveiling these features is not a trivial task. Indeed, data from preliminary *in vitro* experiments are often scarce with respect to the data sizes required for complex ML techniques, which are knowingly data-hungry, especially when the readouts require time-consuming manual sample preparation. To overcome these limitations, we propose to leverage (simple) unsupervised ML strategies and feature importance techniques to learn the engineered variables that allow for distinguishing cell phenotypes based on data gathered in our already described breast cancer *in vitro* models. The resulting combination of breast-specific MPS and (simple) ML models is used to investigate the effect of TME on invasive potential and/or recurrence risk, toward achieving the ultimate goals of this study: (1) uncovering the biomarkers that should be monitored to detect invasive breast cancer cell phenotypes over noninvasive ones for an in-depth characterization and (2) understanding whether these biomarkers depend on the properties of the MPS model. To the best of our knowledge, this approach has not been reported previously, providing a unique and innovative platform for studying such critical cellular processes in a highly physiological and pathological context.

## 2 Experimental Materials and Methods

### 2.1 Data Collection

In this study, we included 12 different tissue-specific microenvironments (Table 1) and used two different human breast cancer cell lines and obtained 24 breast-specific MPSs; Table 2 reports the 16 different cellular responses/outputs measured for each condition and cell line used.

**Table 1:** Breast-Specific MPSs (12) and Corresponding Physical Features Used to Model Healthy and Tumoral States^a^ ^,^ ^b^

MPS	hydrogel (E, kPa)	alginate (% w/v)	gelatin (% w/v)	pHe	fluid-mediated flow/force
1	So-L (1.8 ± 0.2)	1.5	1	7.4	static
2				6.5	static
3				7.4	dynamic
4	So-H (2.4 ± 0.1)	1.5	3	7.4	static
5				6.5	static
6				7.4	dynamic
7	St-L (6.1 ± 0.2)	3	1	7.4	static
8				6.5	static
9				7.4	dynamic
10	St-H (10.1 ± 0.5)	3	3	7.4	static
11				6.5	static
12				7.4	dynamic

a So = soft hydrogel (E <4 kPa, normal); St = stiff hydrogel (E >5 kPa, tumor); L = low gelatin content; H = high gelatin content.

b Alginate-based hydrogels were classified based on their mechanical properties (E, Young’s Modulus) and density (gelatin content). Inclusion of cell-laden hydrogels in a bioreactor was used to transmit forces (dynamic) and compared to traditional cell culture (static). The ph of cell culture medium was buffered to values reported for healthy (pH 7.4.) or tumor (pH 6.5) tissues.

**Table 2:** Experimental Data Used as the Output of Tested MPS Models (*n* = 12; Table 1) for Each Human Breast Cell Line (*m* = 2; i.e., MCF-7, MDA-MB-231)

experimental method	measured data
cell proliferation alamar blue assay	metabolic activity in time (time points: day 1, day 4, day 7, day 14)
breast cancer stem cell marker (B-CSC) flow cytometry analysis	CD44 (MFI), CD24 (MFI), CD44v6 (MFI), CD44+ (%), CD24+ (%), CD44+/CD24– (%), CD44v6+ (%), ALDH+ (%)
epithelial-to-mesenchymal marker flow cytometry analysis	vimentin (MFI), E-cadherin (MFI), vimentin+ (%), E-cadherin+ (%)

#### 2.1.1 Engineered 3D Breast in Vitro Models

##### 2.1.1.1 Human Breast Cells

Human breast adenocarcinoma cell lines MCF7 and MDA-MB-231 were selected and authenticated by the European Collection of Authenticated Cell Cultures (ECACC, operated by Public Health England) prior to use. Cells were routinely cultured in complete medium and were discarded after they reached passage 25 for MDA-MB-231 and passage 50 for MCF-7. In specific, two breast cancer cell lines were chosen to represent opposite ends of the breast “invasive potential” spectrum: the highly invasive triple negative cancer cell line (MDA-MB-231) and less invasive luminal type (MCF-7).

##### 2.1.1.2 3D In Vitro Models

Alginate-based hydrogels characterized in our previous study^15^ were selected based on their elastic modulus (soft vs stiff), density, and gelatin content (low vs high) and to mimic characteristics of normal (soft, low) and cancer breast tissue (stiff) (Table 1). Hydrogels were designed to target Young’s modulus values (also referred to as stiffness) from 1 kPa (normal breast tissue) to 10 kPa (tumor breast tissue); low and high gelatin concentration to mimic either the normal breast tissue or collagenous dense tumor tissue. For 3D cell culture studies, spherical hydrogel beads were prepared gently resuspending cells in alginate precursor solutions (aq) ensuring a homogeneous single cell suspension and at a concentration of 10^6^ cells/mL. Then, single droplets of cells suspended in alginate were ejected from a 25G needle in a sterile cross-linking solution allowing gelation for 10 min at room temperature (RT).

##### 2.1.1.3 Environmental pH

Different medium compositions were used to model pH variations and tailor a normal (i.e., pH 7.4) and a cancer (i.e., pH 6.5) microenvironment. DMEM buffered cell culture media were prepared with HEPES-PIPES.^15^ To ensure maintenance of the target environmental pH, cell culture media were changed every 2 days, with cells cultured in standard conditions (37 °C, 5% CO_2_).

##### 2.1.1.4 Intratumoral Pressure: Bioreactor and Force Transmission

To model force transmission as in TME, interstitial fluid flow of the bioreactor was used to transmit mechanical forces to cells: the Quasi vivo QV500 system (Kirkstall, UK) equipped with the Watson-Marlow 202U peristaltic pump was used as reported in ref 15 and setting a constant flow rate of 500 μL/min. The flow rate was selected based on supplier’s suggestion and using flow rate values already reported in the literature while modeling breast-specific microenvironments.^21^

#### 2.1.2 Cell Viability

Alamar blue assay (Deep Blue Cell Viability Kit 424701, Biolegend) was used to analyze cell proliferation at different time points (i.e., day(s) 1, 4, 7, and 14) without disrupting samples, following manufacturer’s instructions. Data were obtained using the Synergy-2 (Biotek) plate reader (λ_ex_ 530–570 nm/λ_em_ 590–620 nm). All intensity measurements at days 4, 7, and 14 were normalized with their respective readings at day 1. Data were acquired in triplicate on three independent biological experiments.

#### 2.1.3 Biomarker Expression: Flow Cytometry

Marker expression (i.e., CD44, CD44v6, CD24, E-cadherin, vimentin, ALDH) was analyzed by flow cytometry (BD Fortessa X-20) and following the procedure reported by Shah et al.^15^ To exclude dead cells from measurements, cells were incubated with 1 μg/mL of 4′,6-diamidino-2-phenylindole (DAPI) solution in 1 × PBS (5 min, RT) and then washed with 1 × PBS and resuspended in 1 × PBS for further measurements. Data were analyzed with FlowJo software (v10.8.0, BD) and for gate single live cells and obtained measurements on median fluorescence intensity (MFI) and number of cells positive for each marker (%). The gates for ALDH+ (%) cells were sorted based on the DEAB negative control of the respective sample. The median intensity of the marker was normalized by its respective isotype control for every sample and then plotted as an average of *N* = 3 independent experiments.

### 2.2 A Data-Driven Approach toward Profiling Breast Cancer Phenotypes

The available data were initially grouped based on the TME characteristics (Table 1) and preliminarily analyzed by computing their descriptive statistics, i.e., the median, the 25th and 75th percentiles of the available records, and the minimum (0th percentile) and maximum (100th percentile) records excluding outliers (Figure 6B) for a visual representation of the former statistics through boxplots per biological readouts and TME). Two different data-driven paths are then followed to use the measured markers to characterize interphenotype differences (among two cell lines) and intraphenotype (within same cell lines) behaviors, whose main tools and features are described next.

#### 2.2.1 Data Processing Tools

To unveil the peculiarities of the two considered cell phenotypes with respect to features of the microenvironment, we jointly used a set of off-the-shelf tools from statistics and machine learning, namely, the sample Pearson correlation coefficient, *k*-means,^22^ the silhouette index,^23^ and the mutual information score.^24^ Toward formally introducing these tools, we referred to the *j*th marker as *x* ^*j*^, with *j* ∈ {1, ..., *n*} ⊂ *N* being the number of measured markers throughout our experiments, while we indicated the *k*th feature of the microenvironment as *u* ^*k*^, for *k* ∈ {1, ..., *m*} ⊂ *N*. Meanwhile, we referred to the *i*th sample of the *j*th marker available in our data set as *x* ^*ij*^, for *i* = 1, ..., *N* (with *N* ∈ *N* being the cardinality of the available data sets) and with *j* ∈ {1, ..., *n*}. The markers and experimental conditions in each record of our data set are collected into two vectors, respectively, denoted as *x*_*i*_ ∈ *R* ^*n*^ (and often referred to as the feature vector) and *u*_*i*_ ∈ *R* ^*m*^, for *i* = 1, ..., *N*.

According to the previous definitions, the Pearson correlation coefficient was used to quantify the strength and direction of the (linear) relations between two markers or a marker and a microenvironment condition based on the available measured markers. Formally, when looking at markers’ correlations, such score can be defined as described in eq 1:

1 $$\rho_{j,h}^{x}=\frac{\sum_{i=1}^{N}\left(x_{i}^{j}-{\mathrm{x̲}}^{j}\right)\left(x_{i}^{h}-{\mathrm{x̲}}^{h}\right)}{\sqrt{\sum_{i=1}^{N}{\left(x_{i}^{j}-{\mathrm{x̲}}^{j}\right)}^{2}}\sqrt{\sum_{i=1}^{N}{\left(x_{i}^{h}-{\mathrm{x̲}}^{h}\right)}^{2}}}\in \left[0,1\right],,j,h\in \left\{1,...,n\right\},j\neq h$$

where ${\mathrm{x̲}}^{j}$ and ${\mathrm{x̲}}^{h}$ are, respectively, defined as ${\mathrm{x̲}}^{j}=\frac{1}{N}\sum_{i=1}^{N}x_{i}^{j}$ and ${\mathrm{x̲}}^{h}=\frac{1}{N}\sum_{i=1}^{N}x_{i}^{h}$ , with *j*,*h* ∈ {1, ..., *n*}, *j* ≠ *h*. The indicator is instead defined in eq 2:

2 $$\rho_{j,k}^{x,u}=\frac{\sum_{i=1}^{N}\left(x_{i}^{j}-{\mathrm{x̲}}^{j}\right)\left(u_{i}^{k}-{\mathrm{u̲}}^{k}\right)}{\sqrt{\sum_{i=1}^{N}{\left(x_{i}^{j}-{\mathrm{x̲}}^{j}\right)}^{2}}\sqrt{\sum_{i=1}^{N}{\left(u_{i}^{k}-{\mathrm{u̲}}^{k}\right)}^{2}}}\in \left[0,1\right],,j\in \left\{1,...,n\right\},k\in \left\{1,...,m\right\}$$

when evaluating the correlation between markers and microenvironment features, with ${\mathrm{u̲}}^{k}=\frac{1}{N}\sum_{i=1}^{N}u_{i}^{k}$ for *k* ∈ {1, ..., *m*}. In this work, these indicators were used to disclose correlations between the measured markers as well as those between them and the microenvironment features when looking at the single-cell phenotypes.

Meanwhile, *k*-means was used to group the available markers into a predefined number of clusters *G* ∈ *N* by looking at central trends in the data, toward discovering the ones that were relevant for distinguishing between phenotypes and the existence of different intracluster behaviors. Starting from an initial guess of the groups’ means μ_*g*_^(0)^ ∈ *R* ^*n*^ at the 0th iteration, for *g* = 1, ..., *G*, *k*-mean unfolds by recursively carrying out two steps. At each iteration *q* ≥ 0, with *q* ∈ *N*, each record was first assigned to a cluster based on its Euclidean distance from the current mean, i.e., each group was constructed according to the logic described in eq 3:

3 $$C_{g}^{\left(q\right)}=\left\{x_{i}:{∥x_{i}-\mu_{g}^{\left(q\right)}∥}_{2}\leq {∥x_{i}-\mu_{l}^{\left(q\right)}∥}_{2},,1\leq l\leq G,g\neq l\right\},g=1,...,G$$

4 $$\mu_{g}^{\left(q+1\right)}=\frac{1}{\left|C_{g}^{\left(q\right)}\right|}\sum_{x_{i}\in C_{g}^{\left(q\right)}}x_{i}$$

Once the groups were created, the clusters’ means (also called centroids) were then updated as described in eq 4, where *|C*_*g*_(*q*)*|*denotes the cardinality of the *g*th cluster at the *q*th iteration. This iterative procedure was carried out in an unsupervised way (i.e., without requiring the data points to be preassigned) until a termination criterion was satisfied, e.g., points’ assignments do not change over two consecutive iterations. Note that, according to the grouping logic in eq 3, ambiguous points (namely, those that can be assigned to multiple clusters) were assigned to one cluster only.

Despite the intuitiveness of *k*-means, this algorithm rests on the assumption that the number of groups *G* into which the available features have to be partitioned is prefixed. While in our setting this is true when distinguishing between cell phenotypes, this was not the case when trying to unveil different behaviors within the same cell line. To determine the number of clusters in this second scenario, we used the silhouette index in eq 5, where *C*_*g*_ is the *g*th cluster after the termination of *k*-means and *s*(*x*_*i*_) is described in eq 6:

5 $$S=\frac{1}{\left|C_{g}\right|}\sum_{x_{i}\in C_{g}}s\left(x_{i}\right)$$

$$s\left(x_{i}\right)=\frac{b_{i}-a_{i}}{\max\left({a_{i},b}_{i}\right)},,\mathrm{for}\;x_{i}\in C_{g},\mathrm{if}\;\left|C_{g}\right|>1\;\mathrm{and}\;s\left(x_{i}\right)=0,\mathrm{if}\;\left|C_{g}\right|=1$$ 6

with *a*_*i*_ and *b*_*i*_, respectively, used to assess a cluster’s cohesion and its separation from the others, defined as

7 $$a_{i}=\frac{1}{\left|C_{g}\right|-1}\sum_{x_{p}\in C_{g},p\neq i}{∥x_{i}-x_{p}∥}_{2},,b_{i}=\min_{l\neq g}\frac{1}{\left|C_{l}\right|}\sum_{x_{v}\in C_{l}}{∥x_{i}-x_{v}∥}_{2},,i=1,...,N$$

Note that the silhouette index *S* in eq 5 ultimately satisfied *S* ∈ [–1, 1], with values of *S* close to 1 indicating that samples were coherent and well separated while *S* close to 0 or being negative denoting possible overlaps between clusters or a non-negligible misclassification rate, respectively. Note that, apart from selecting the number of behaviors recognizable from the available markers, in our approach, we further used the silhouette index as an indicator of the clusters’ quality, ultimately guiding the choice of the markers that allowed the discrimination between cell phenotypes. Apart from using the silhouette index, the selection of relevant markers for the characterization of cell phenotypes was also performed by using the mutual information score, estimating the mutual information between each marker and the grouping labels available when distinguishing between cell phenotypes and not used by *k*-means for clustering.

#### 2.2.2 A Bird’s Eye View on Intraphenotype Relationships and Interphenotype Differences

As an initial phase to understand links between markers, microenvironment conditions, and cell phenotypes, our data-driven pipeline consisted of a preliminary step where Pearson’s correlation coefficients between markers (see eq 1) and that between the markers and the different features of the microenvironment were estimated for each cell line separately. By inspecting them, these coefficients provided initial insights into differences in the relations between markers and microenvironment conditions among different cell lines while indicating which markers were more likely to react to changes in a specific microenvironment characteristic when considering a single cell line. Nonetheless, looking at Pearson’s correlation was not sufficient to have a quantitative and concrete approach to simplify the experimental scheme and determine the factors that were necessary to differentiate between metastatic and recurrent features of MDA-MB-231 compared to the nonrecurrent ones of MCF-7.

#### 2.2.3 Are There Any Discernible Intraphenotype Behaviors Depending on the Microenvironment Condition?

Apart from distinguishing among cell phenotypes, further information on intraphenotype behaviors driven by the different microenvironment conditions can be key to simplifying experiment design (as well as making it more sustainable) while enabling a deeper understanding of the interactions between the microenvironment and the cells. To this end, *k*-means was exploited to determine whether distinct behaviors could be detected within each cell line depending on microenvironment conditions, using the measured markers as features for clustering purposes. In this case, the number of clusters could not, however, be selected a priori. Therefore, in detecting behaviors within the same group of cells, we separated the markers into an increasing number of clusters up to a maximum, exploiting the silhouette index (see eq 5) to evaluate the quality of separation and then decide the amount of different behaviors (if any) detectable within a single cell line. In particular, the number of clusters was ultimately chosen as the one leading to the number of groups that causes the least drop of the silhouette score, picking the minimal number of groups if two tested values lead to comparable silhouette scores. This design choice was made to search for a trade-off between accuracy (i.e., limited drops on the attained silhouette index’s values) and number of identified behaviors. Note that, in this case, no pre-existing labels were available as ground truth to characterize intraphenotype behaviors and, thus, feature selection (i.e., unveiling unneeded markers) could only be performed by trial and error, removing one feature at a time and evaluating changes in the resulting silhouette score.

#### 2.2.4 Toward Sustainable Profiling of the Invasive Potential of Breast Cancer Cells

As already mentioned, the two cell lines considered in this study (MCF-7 and MDA-MB-231) are associated with different invasive potentials. Understanding which experimental conditions (see Table 1) and measured markers (Table 2) allowed one to distinguish between the two cell lines would be key to informing future experiments and guide the analysis of other cell phenotypes. To this end, by using the techniques introduced in Section 2.2.1, we investigated whether all of the measured markers (Table 2) were needed to distinguish between the responses of MCF-7 and MDA-MB-231, and if there existed also a relationship between the TMEs and the relative importance of the markers in performing such a distinction. Toward grasping such an understanding, we proposed the use of the data-driven pipeline, as schematized in Figure 4, involving three main stages. Initially, *k*-means (see Section 2.2.1) was used to cluster the available features (i.e., the recorded markers) into two groups by fixing all microenvironment conditions but one. The silhouette index (see eq 5) was then employed to evaluate the separability between the obtained groups, hence assessing the quality of the results achieved via the first unsupervised clustering routine. Note that this first phase did not de facto exploit our prior knowledge of the actual cell line (i.e., the label) each data record was associated with, in turn, allowing us to use all the available (yet scarce from a ML perspective) samples to carry out *k*-means. Nonetheless, the knowledge of the actual cell line can already be used to check the quality of the grouping one blindly obtains by employing this clustering technique using all the available markers, already giving an insight into whether they were all needed to distinguish between MCF-7 and MDA-MB-231. Our prior knowledge of the actual cell line each set of markers is associated with is instead used in the second step of our procedure, i.e., feature selection. To this end, an estimate of the mutual information between each available marker and the actual label (i.e., invasive/noninvasive cell phenotype) was employed to rank the available features, toward unveiling those markers that are likely unnecessary to distinguish between cell lines. Indeed, the mutual information score measures the mutual dependence between a feature and the label by computing the difference between the entropy of the features and the conditional entropy of the features given the labels, or, equivalently, the difference between the entropy of the labels and their conditional entropy with respect to the features.^25,26^ This additional step allows for discarding noninformative markers by thresholding the mutual information. Note that, for now, thresholding is performed manually by retaining only those features whose mutual information score is (strictly) greater than 0.65. While this choice has proven effective in all our tests, we aim to explore strategies to automatize the thresholding procedure in the future.

The resulting reduced set of features is ultimately clustered again through K-means, and its outcome is validated quantitatively by looking at the silhouette index and comparing the label attributed to the data in an unsupervised fashion with the actual ones. Note that, to grasp the implication of the different microenvironment conditions (i.e., pH, perfusion, matrix stiffness, and composition), this procedure is carried out by fixing one microenvironment condition at a time and not explicitly considering any of the remaining microenvironment characteristics among the features to be clustered via *k*-means.

## 3 Results

### 3.1 Intraphenotype Descriptive Statistics and Correlation Plots

To visualize the correlations among all the parameters of the engineered *in vitro* system, we first plotted the measured data of cellular phenotypes and the microenvironmental conditions to create two separate Pearson correlation matrices, one for each cell line (Figure 2). The complexity of the plot indicates that there are numerous factors to consider in comparing the two cell lines. Notably, there are significant differences as well as similarities in the correlation patterns among the MDA-MB-231 and MCF-7 cells. For instance, in MCF-7 cells, E-cad (both median fluorescence intensity and + %) is positively correlated with pH and perfusion, whereas in MDA-MB-231, E-cad is positively correlated with mean hydrogel stiffness. In both the cell lines with respect to B-CSC marker expression, ALDH+ (epithelial type CSCs) is negatively correlated to increase in perfusion and pH, but it is positively correlated to stiffness. On the other hand, CD44+/CD24– (mesenchymal-type CSCs) is always positively correlated to perfusion. This aligns with previous in vivo studies that mention CD44+/CD24– cells are localized at the tumor boundary (known for high perfusion rates), while ALDH + cells reside in the tumor core (known for an acidic microenvironment).^19,27^ It is known that B–CSCs exhibit plasticity, transitioning between E-CSCs and M-CSCs,^19^ and the TME could potentially influence this transition. Indeed, extracellular acidic pH is known to enhance reactive oxygen species (ROS) formation in cancer cells independently^28^ and the presence or treatment of B–CSCs with ROS (H_2_O_2_) is shown to induce transition to E-BCSCs as they are endowed with elevated NRF2 antioxidant defenses compared to M-BCSCs.^29^ In case of interstitial fluid flow, it is known to increase stem cell invasion via CD44-mediated mechanisms in glioblastoma.^30^ Our observations alongside these studies reinforce the importance of a physicochemical microenvironment which irrespective of cell lines can affect possible spatial distribution of E-CSCs in the tumor core as compared to M-CSCs at the tumor boundary.

**Figure 2:**
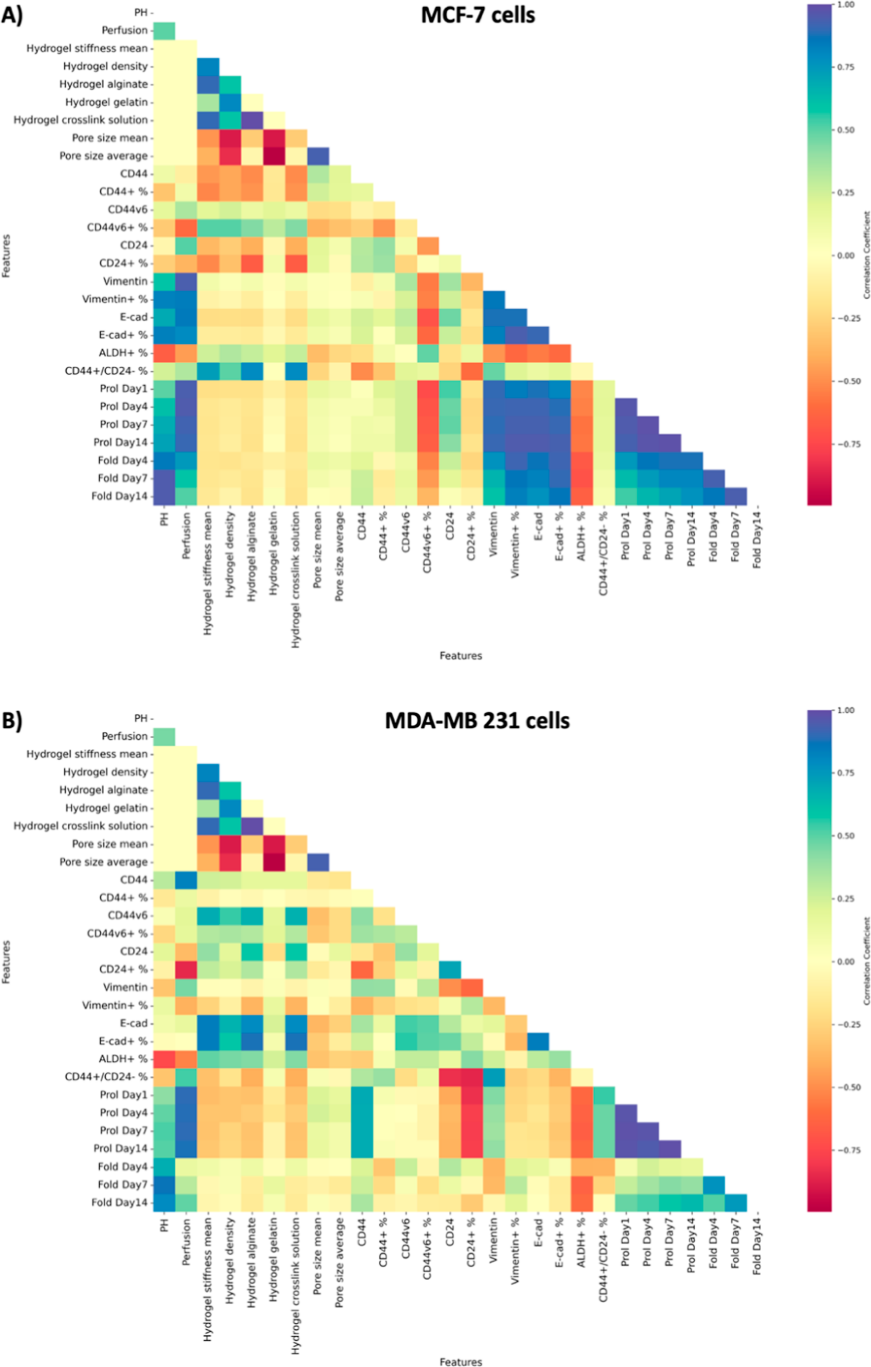
Correlation plot of all features of the breast cancer MPS model: Pearson correlation coefficients computed among microenvironmental conditions of the MPS model and measured cellular markers, plotting possible linear correlations of either positive (blue), neutral (yellow), or negative (red) correlation in (A) noninvasive MCF-7 cells and (B) invasive MDA-MB-231 cells.

Within the same cell line, the varied influence of biophysical parameters is better compared by using the correlation plot. For MCF-7, pH and perfusion show a greater correlation with changes (both positive and negative) in cellular phenotypes than the hydrogel properties. In MDA-MB-231, most parameters affect cellular phenotypes. However, in both cell lines, the hydrogel gelatin content shows little-to-no correlation with phenotypic changes compared to hydrogel stiffness, implicating that this parameter could be removed in future experimentation.

In summary, the correlation plot may help in providing a bird’s eye view of the data for visual comparison. It helped in discerning certain patterns with marker expression compared with the two cell lines. In terms of experimental design, the correlation plot aided in visualizing certain biophysical parameters that could be influential in bringing about changes in the cellular phenotype. However, it does not provide a robust, quantitative, and concrete approach to simplify the experimental scheme and determine which factors would be absolutely necessary to differentiate among the metastatic and recurrent features of MDA-MB-231 compared to the nonrecurrent ones of MCF-7.

Next, to recognize patterns of cell behavior in differing microenvironments, the cellular outputs or features (proliferation, EMT, and B-CSC marker expression) from all microenvironments were clustered for each cell line using *k*-means. The optimal number of clusters was decided using the silhouette index, with the clustering that yielded the highest silhouette score being chosen (Figure SI 1). As evident for both MDA-MB-231 and MCF-7 cell lines, the highest silhouette index was when *k* = 2 clusters were formed. To visualize this clustering, among the 16 measured features, we first plotted three features, namely, proliferation (day 7), CD44+/CD24–, and ALDH+ in different microenvironments where each data point was colored based on their classification in two clusters (as determined previously with all the features) (Figure 3).

**Figure 3:**
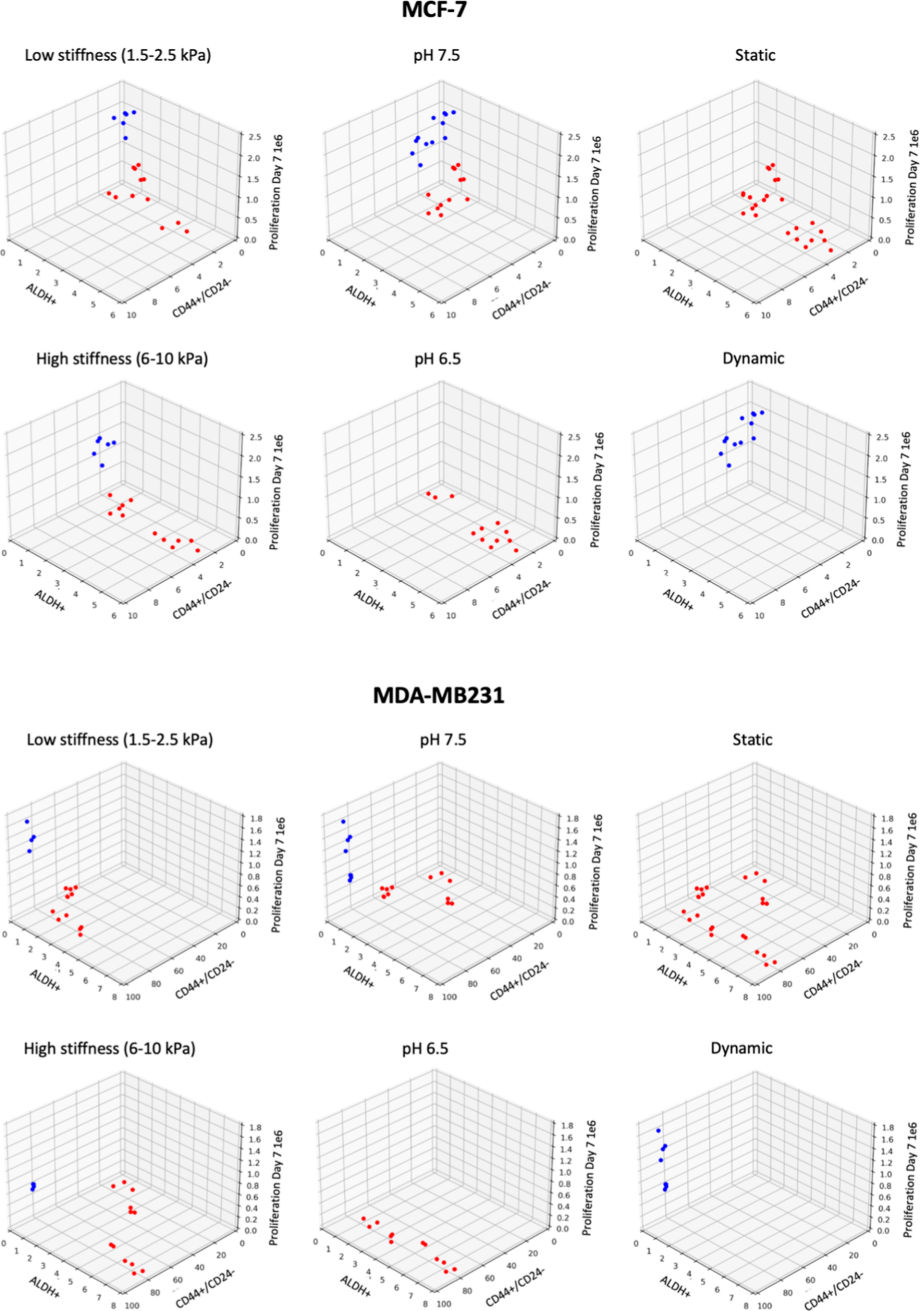
k-Means-based clustering of cellular phenotypes in different microenvironments: Dot plot depicting ALDH+ (%), CD44+/CD24– (%), and proliferation (day 7) in low/high stiffness, pH 6.5/pH 7.4 and static/dynamic flow conditions for MCF-7 cells (top) and MDA-MB-231 (bottom). Red and blue depict two cluster population based on *k*-means clustering of all cellular phenotypes in different microenvironments.

Based on the clusters formed, it is evident that cell lines respond differently across some microenvironments yet crucially also exhibit similarities in some. This is best exemplified by the single clusters present when cell lines are cultured in pH 6.5, static and dynamic conditions, suggesting these conditions create mostly homogeneous population concerning these cellular phenotypes. We also observe a consistent inverse relationship of high proliferation of bulk tumor cells with low ALDH + populations (blue cluster). This is noteworthy because ALDH + cells, known for their tumor-initiating capacity,^31^ typically increase in the presence of cytotoxic or cytostatic stressors, such as those induced by tamoxifen^32^ or ROS.^29^ This effect is not limited to ALDH, as reports also suggest the increase of CD44+/CD24– cells in case of drug-related cytotoxicity and tumor cell death.^33^ It is interesting, however, that we observe the converse (i.e., high proliferation correlating with low ALDH + population) only for E-CSCs. This could be supported by the hypothesis that ALDH + populations are mostly quiescent in non-cytotoxic conditions.^34^ These observations highlight the need for further research into the growth dynamics of bulk tumor cells and their relationship with CSC populations when exposed to environmental stressors.

As such, dynamic conditions create higher proliferating and low ALDH + populations, whereas pH 6.5 creates low proliferating and high ALDH + populations. As mentioned before, tumor core that is characterized by low pH is observed to have the presence of high ALDH+ (E-CSCs) populations in vivo,^19^ which is also apparent in our analysis irrespective of the cell line. In contrast, pH 6.5 and dynamic conditions maintain CD44+/CD24– at higher levels in MDA-MB-231 but not in MCF-7, creating a distinction in their behavior. This analysis suggests that for streamlining future experiments, where we can differentiate between MCF-7 and MDA-MB-231, a combination of “microenvironment” and “cellular phenotypes” will have to be considered. However, with these graphs, it is difficult to visualize more than three parameters at a time, which leads to incomplete analysis. At this stage, dimensionality reduction methods may help in visualization by combining all of the parameters to create new component variables. However, this can reduce the interpretability of contribution from individual original variables or features and defeat the purpose of data-informed experiment streamlining based on high impact features. Overall, this analysis, while providing some insights in cell behavior, still fails to provide a robust and scalable method that can be easily adopted for other bioengineering setups or translated to larger clinical data sets.

### 3.2 Data Processing Pipelines toward Profiling the Breast Cancer Invasive Potential

To develop a quantitative approach that could inform on features important to differentiate between MDA-MB-231 and MCF-7 profiles, we utilized a data processing pipeline based on unsupervised *k*-means clustering, feature extraction, and feature selection applied with all the parameters of the MPS model (Figure 4). However, for this method, cellular data from both the cell lines was used together for clustering. Briefly, output parameters, representing distinct cellular phenotypes, were clustered for each microenvironmental input parameters, with silhouette indexes used to assess the initial unsupervised cluster separation. By comparing to actual labels, cellular phenotypes were ranked based on their contribution in distinguishing MDA-MB-231 and MCF-7 profiles. Eventually, low-ranked features were removed, and clustering was re-evaluated to validate differentiation accuracy (Figures 4, 5). By using this method, we were able to extract features that were absolutely necessary to distinguish MDA-MB-231 versus MCF-7 in each microenvironmental condition. The output/features from each microenvironmental condition, namely, the pH (pH 7.4 or pH 6.4) (Figure 5), perfusion status (static or dynamic), matrix stiffness (hydrogels So-L, So-H, St-L, St-H), or matrix composition (Alginate or gelatin content) were processed with this pipeline and the features were ranked (Supporting Information, Figures SI 2 and Table SI 1). Interestingly, each microenvironment had a different set of features that were important for the distinction between the two cell lines (Figure SI 2). For example, pH 6.5, dynamic perfusion, and soft matrixes resulted in the highest number of important features necessary for this distinction, suggesting that these microenvironments may help in better isolating the invasive phenotypes from noninvasive ones. A confusion matrix table that showcases how well a machine learning model performs by comparing predicted values to actual values in a data set was used to assess the accuracy of the ML classification model after feature reduction for each condition (Figure 5). As confirmed by this matrix, both false positives and false negatives become zero after feature extraction and reduction. Overall, this data pipeline streamlines the experimental process and aids in identifying which features distinguish between invasive and noninvasive profiles if a specific microenvironmental condition is selected.

**Figure 4:**
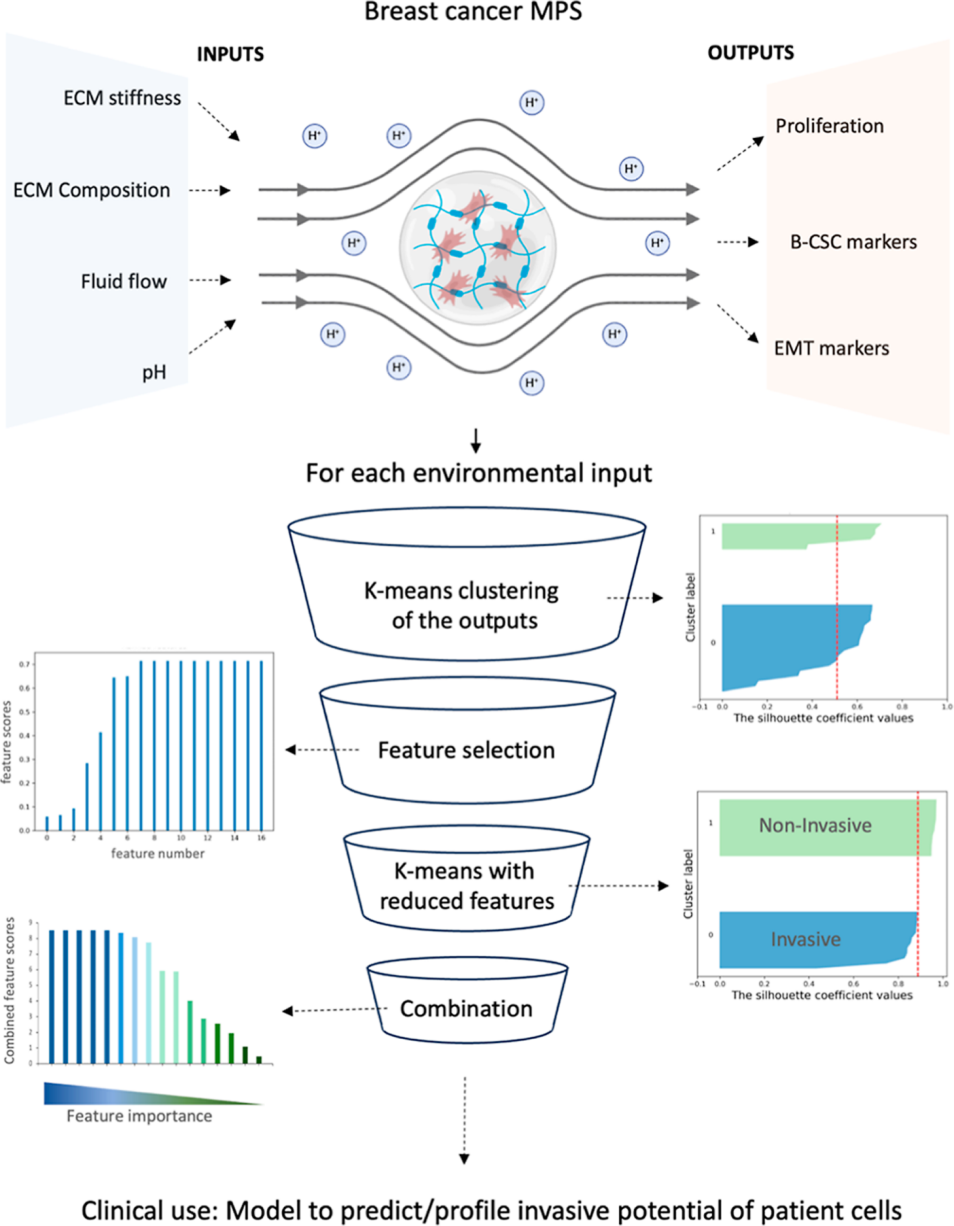
Schematic representation of the digital tools used for profiling invasive and recurrent behaviors in breast cancer cells. ML-based data pipeline outlining steps to profile invasive MDA-MB-231 and noninvasive MCF-7 cells by (1) pooling all cellular phenotypes of both cell lines in each microenvironmental condition, (2) performing unsupervised *k*-means clustering and keeping the number of cluster constant (*K* = 2), (3) ranking features/cellular phenotypes based on the alignment of the features with the true label, (4) performing *k*-means again with selected and reduced set of features and confirming with the silhouette index to plot clusters with true labels, and (5) combining feature scores from all microenvironments to rank the most important features useful in distinction of invasive phenotypes.

**Figure 5:**
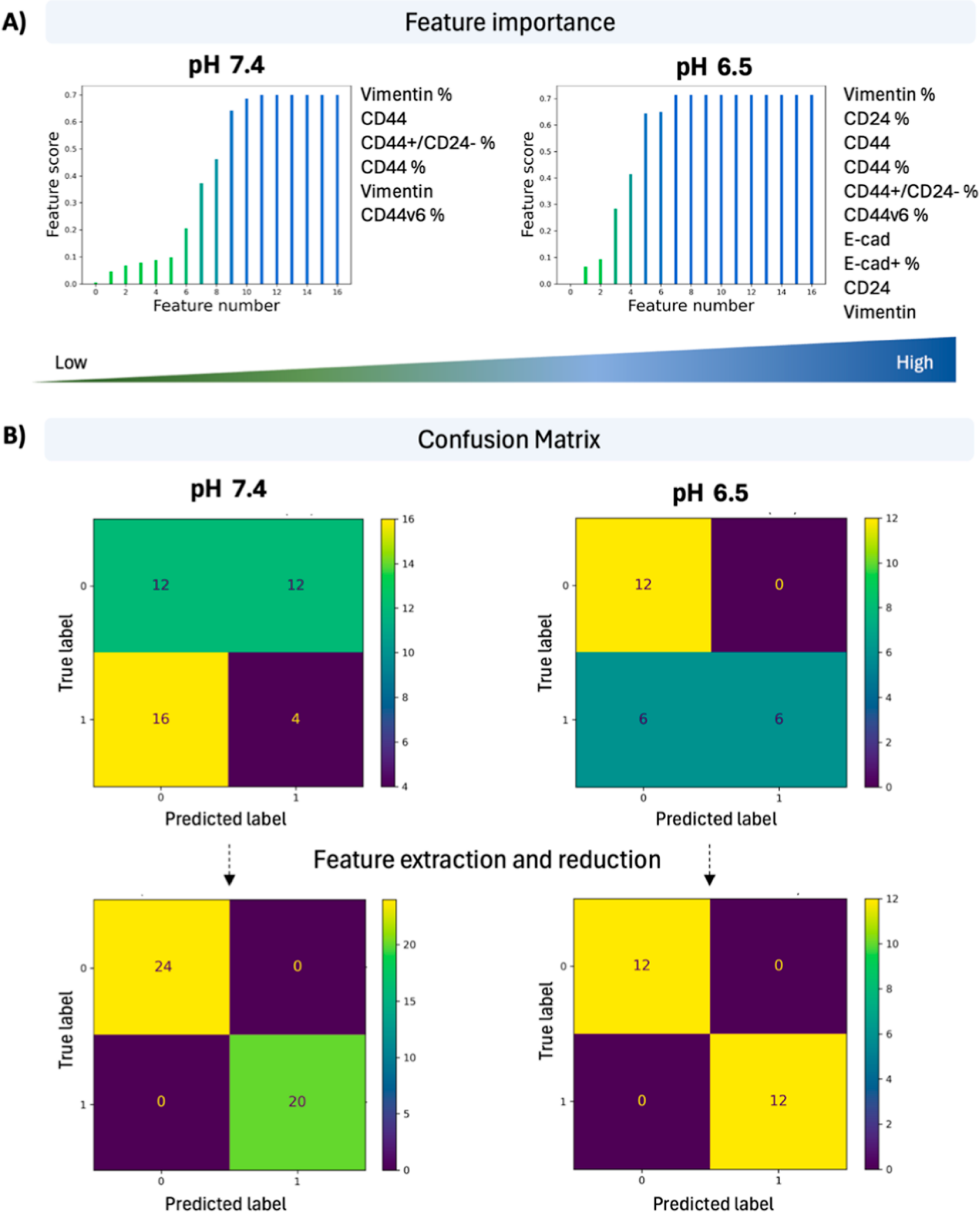
Ranked feature scores of cellular phenotypes in a microenvironmental input: (A) Example of feature score plot where features/cellular phenotypes are ranked from low to high (left) and the list of most important features (right) when BC cells are cultured in pH 7.4 and pH 6.5. (B) Confusion matrix showcasing true positive (upper left), false negative (lower left), false positive (upper right), and true negative labels (lower right) before and after feature extraction in pH 7.4 and pH 6.5 conditions.

### 3.3 Unveiling the Markers Needed to Detect Invasive Cell Phenotypes

To determine which features were consistently ranked as important across all microenvironmental conditions, we aggregated the feature scores of all of the outputs represented in Supporting Information Table SI 1. The features that frequently scored highest were CD44, CD44^+^ (%), CD44v6, CD44v6 (%), Vimentin, and Vimentin^+^ (%), while the lowest-scoring features were cellular proliferation and ALDH^+^ (%) (Figure 6). Histograms revealed that the expression of high-importance features like Vimentin was consistently lower in MCF-7 or noninvasive profiles, even under tumorigenic conditions such as low pH, high stiffness, and dynamic environments, whereas in MDA-MB-231, Vimentin^+^ population was always high. For features of moderate importance, such as CD24^+^, MDA-MB-231 displayed variable levels depending on the microenvironment, sometimes indistinguishable from MCF-7. Low-importance features like ALDH^+^ showed lower levels in both cell lines, with no significant differences among them across all microenvironments. The histogram/box-plot analysis supports the conclusions from our data pipeline, which suggests that this approach can provide a robust quantitative methodology for feature selection and develop a data-driven sustainable approach to narrow down experimental parameters. It was observed that mesenchymal markers and the CD44^+^/CD24^–^ status were more important than the epithelial marker (E-cadherin) and ALDH^+^ status. This indicates that within the CSC population, mesenchymal CSCs (M-CSCs) are better predictors of a recurrent phenotype than epithelial CSCs (E-CSCs).

**Figure 6:**
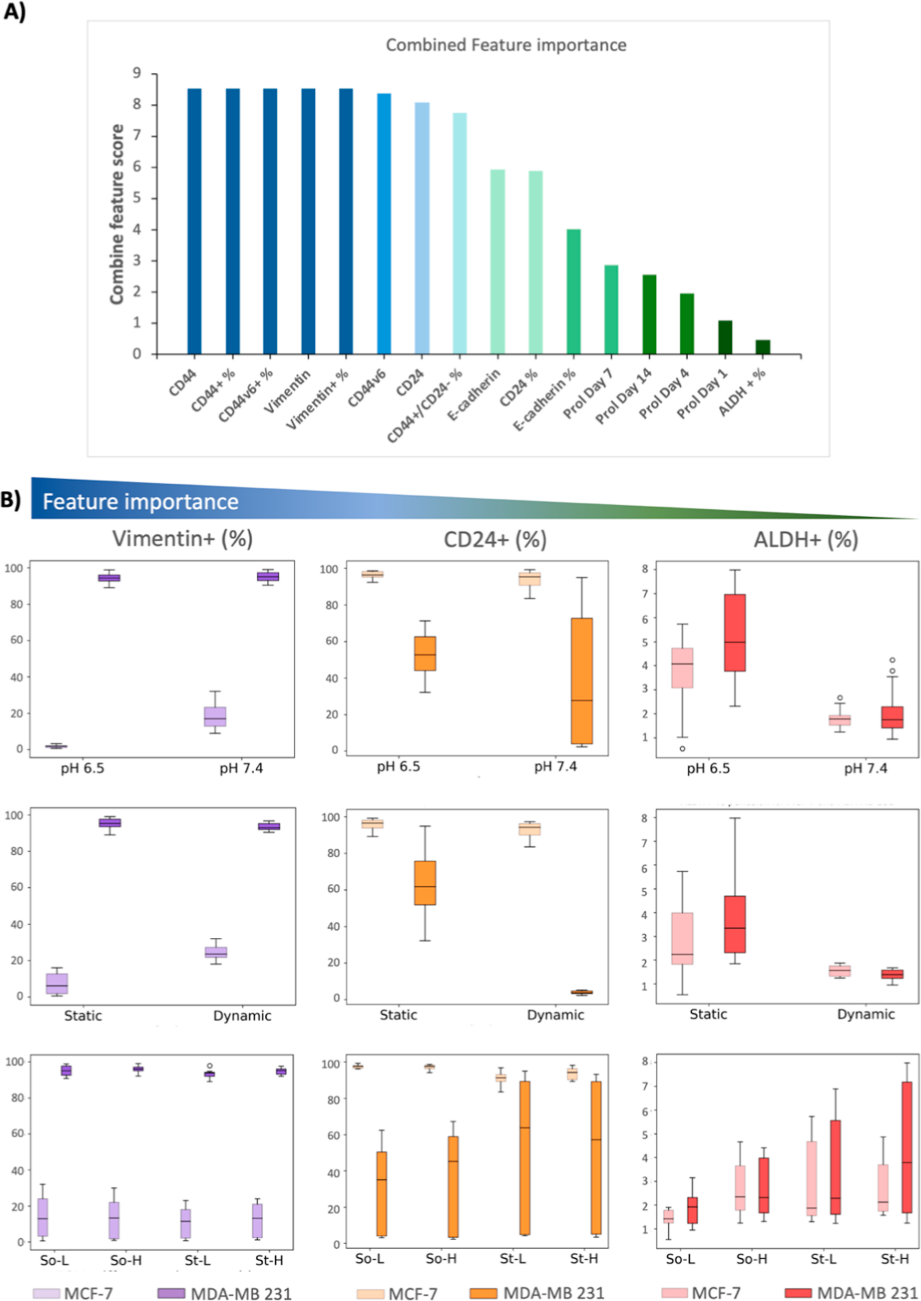
Combined feature importance of cellular phenotypes: (A) combined feature score of the cellular phenotypes in all the microenvironments, arranged from the highest to the lowest. (B) Bar plot representation of Vimentin (%), high importance; CD24 (%), intermediate importance; and ALDH (%), low importance in different microenvironments for noninvasive MCF-7 (light shade) and invasive MDA-MB-231 (darker shade).

## 4 Discussion

MPS models integrate human cells with engineered microenvironments to mimic key physiological responses, providing a more dynamic and realistic setting than conventional cell culture systems. MPS aims to simulate the mechanical, chemical, and biological stimuli that tissues experience *in vivo*, making them powerful tools for drug testing, disease modeling, and personalized medicine.^35–37^ However, the complexity of MPS models lies in their intricate design and functionality. Recreating the microenvironment, ensuring proper cell–cell and cell–matrix interactions, and maintaining fluid flow and other mechanical cues are technically demanding for high-throughput experiments. Integrating multiple variables for studying systemic responses can prove a challenge in standardization, scalability, and reproducibility, as it leads to complex experimental settings and loss of quick data interpretation. There is no fixed definition for the optimal balance between the model complexity and its accuracy or efficacy in delivering relevant *in vivo* physiology insights for a specific application. This holds true for most *in vitro* campaigns where preliminary experiments often include or exclude specific variables arbitrarily, without a rigorous rationale. A more systematic approach to variable selection would enhance the quality and impact of *in vitro* research, fostering more dependable data that can be confidently built upon in other studies.

In this work, a previously used breast cancer MPS model that mimics various physicochemical aspects of the primary tumor microenvironment was used together with the high dimensional experimental data from various biological, mechanical, and chemical interactions in two cell lines. We selected 12 different microenvironments that mimicked combinations of the normal breast tissue (e.g., soft hydrogel, pH 7.4, and static) and tumor breast tissue microenvironments (e.g., stiff hydrogel, pH 6.5, and dynamic). This ensured that we recorded potential cellular phenotypes in changing microenvironments and not just snapshots that are usually measured as markers of disease progression. We measured proliferation, EMT, and B-CSC markers as these are all knowingly playing a role in breast cancer progression, especially in metastatic invasion which is the cause of most cancer-related deaths.^38,39^ However, with 12 different microenvironments and 16 phenotypic/marker readouts, it was difficult to compare the consequent multidimensional data and to draw conclusions about which experimental settings brought out distinct behavioral patterns of these cell lines.

ML algorithms can help to cope with high-dimensional data sets efficiently, identifying patterns that may help in optimizing experimental conditions and, thus, tests which are otherwise time-consuming. In most preliminary bioengineering setups, experiments are performed with trial and error, wherein variations are introduced in the *in vitro* model and preliminary readouts are manually measured. Although the data is not extensive, it still has high dimensions, thus making it difficult to manually analyze them while having a comprehensive understanding of the information embedded in the data. In turn, this makes it harder to use these insights to inform future directions with experimental schemes. In this study, we have explored machine learning pipelines based on an unsupervised *k*-means approach, along with feature extraction to inform on the most important features of our breast cancer MPS model that differentiate invasive MDA-MB-231 phenotypes from noninvasive MCF-7. Unlike extensive multiomics data or patient-centric medical/clinical data,^40^ preliminary bioengineering experiments typically involve less extensive data, preventing the use of more complex techniques due to the risk of overfitting. The proposed procedure allows for evaluation by leveraging all the available information within the data set, without using dimensionality reduction techniques like Principal Component Analysis (PCA) and Uniform Manifold Approximation and Projection (UMAP). While PCA/UMPAP provides a systematic way to visualize extensive high dimensional data in a lower dimensional space, they may hamper the interpretability of the data analysis’ outcomes. This is because reducing dimensionality can sometimes obscure subtle yet important biological variations or introduce artificial relationships in less extensive data sets, making it harder to directly interpret the underlying biological meaning of the data. While we intend to investigate alternative ML approaches tailored to our scenarios and compare their performance to the proposed pipeline in future works, the data set at our disposal is rather small; therefore, we decided to use only off-the-shelf machine learning tools for our analysis. In particular, to rank and select important features, we evaluated the mutual information between the markers and the labels indicating the cell line associated with each data sample. Meanwhile, *k*-means is only used to assess whether our capability of correctly clustering the data samples enhances, deteriorates, or does not change by removing the features that are deemed not important according to the mutual information.

In fact, with unsupervised *k*-means clustering and feature extraction, we first observed that each microenvironment had a different set of important features that distinguished between the two cell lines. Some microenvironments like acidic pH, dynamic environment, and soft matrix created more differentiating features between invasive and noninvasive phenotypes suggesting that these micro environmental conditions can be further used to validate data with other cell lines and patient samples (Figures 4, 5). Overall, through this data-driven approach, mesenchymal markers such as Vimentin, CD44, and CD44v6 were quantified to be the most important features for distinguishing cell phenotypes (Figure 6). In our experimental setting, this emphasizes that even in differing microenvironments of the primary tumor, M-CSCs and mesenchymal phenotype could be a better discerning factor for invasion and recurrence. In fact, Liu et al. found CD44^+^/CD24^–^ populations were thrice as invasive as ALDH^+^ populations in the TNBC cell line SUM149.^19^ While ALDH^+^ populations had significantly higher tumor initiating capacity than CD44^+^/CD24^–^ cells in TNBC patient-derived xenograft (PDX) models.^31^ This observation does not suggest that M-CSCs are more critical than E-CSCs; rather, it indicates that each cell type possesses distinct functions. Here, the M-CSC population and mesenchymal characteristics likely served as a distinguishing factor, given that MDA-MB-231 cells could adapt to become both M-CSCs and E-CSCs depending on their microenvironment, while MCF-7 cells did not exhibit a comparable increase in mesenchymal features deemed important for invasion.^41^ Ultimately, these findings reinforce the crucial concept that tumor cell plasticity is a key driver of both metastasis and cancer recurrence. This cellular adaptability enables tumors to navigate the complex challenges of spreading throughout the body and resisting therapies, directly contributing to the aggressive nature of the disease. Inclusion of other subtype specific cell lines will bring forward a more relevant list of important features encompassing plasticity across the known spectrum of cell lines. The ML model could further be consolidated by training on extensive data from patient-derived cells (PDCs) of known recurrence and metastasis status in breast MPS models. Compared to cell lines, primary cells can capture patient and subtype specific variations in terms of genetic, epigenetic, and phenotypic diversity. For instance, PDCs from patients with known rapid recurrence or metastatic disease will display specific signatures in changing microenvironments, enabling better risk stratification especially among same subtypes that exhibit recurrence ranging from 5 to 25 years post initial diagnosis.^4^

Thanks to the relative simplicity of the employed tools, the same “blue-print” of data pipeline can be used to investigate and integrate parameters from future experiments as well as other preliminary biology experiments. These can include additional cellular phenotypes/markers as well as patients’ biopsies (either ECM or cells, or both) to translate findings quickly to the clinical setting. With this pipeline, we provide a data-driven approach to look at the data generated by MPS models and a better way to dissect cell–material interaction. This unique combination of bioengineering, tumor biology, and ML could further help in creating sustainable experimental schemes for developing prognostic tools and personalized therapeutics.

## 5 Conclusions

Identifying the relationships between biological readouts and the different TMEs, as well as their likelihood to impact the cell phenotype, is crucial when using patient-derived cells to predict therapeutic outcomes through *in vitro* experiments. This information is key for the design of specific MPSs and sustainable experimental campaigns focusing on the identification of relevant experimental conditions that not only result in time and resource savings but mainly increase the impact of the research.

Toward the implementation of NAMs (New Approach Methodologies) for the assessment of pathophysiology and specifically in cancer development, we developed a new combination of engineered breast-specific MPSs and ML models that represents a first step toward a data-guided outlook on cell/material interactions.

This novel approach combining MPS and ML paves the way for nonanimal-based methodologies in understanding the interplay between TME features and cancer-related biological onsets with a sustainable-by-design approach. The quantitative indications on the TME impact on cancer cell behavior provided by our approach can ultimately steer future experiments, making in vitro approaches more sustainable. At the same time, such an understanding may be helpful at a clinical level in guiding the drug design of cancer therapies.

Due to the straightforward nature of the tools used, the same data pipeline framework can be applied to analyze and incorporate parameters from future clinical experiments and/or early stage biological studies to facilitate the rapid translation of findings into clinical applications.

## Supplementary Material



## Notes

Silhouette plots with different cluster sizes, feature importance ranked under all environmental conditions, and list of all the ranked features (PDF)
