# Supplementary material for: Data-Driven Sustainable In Vitro Campaigns to Decipher Invasive Breast Cancer Features
Source: ACS Biomater Sci Eng. 2025 Jul 25;11(8):5107–21. doi: 10.1021/acsbiomaterials.5c00731 (PMC12370173; doi:10.1021/acsbiomaterials.5c00731)
Supplement: Supplementary file 1 [file ab5c00731_si_001.pdf]

Data-driven sustainable in vitro campaigns to decipher invasive breast cancer features

Lekha Shah[1], Valentina Breschi[2,*], Annalisa Tirella[1,*]

[1] Department of Industrial Engineering and BIOtech Research Center, University of Trento, 38123 Trento, Italy

[2] Eindhoven University of Technology, Electrical Engineering Department, De Zaale, 5600MB, Eindhoven, The Nederlands

* Corresponding authors: v.breschi@tue.nl; annalisa.tirella@unitn.it

SUPPLEMENTARY INFORMATION

SI.1 Silhouette plots

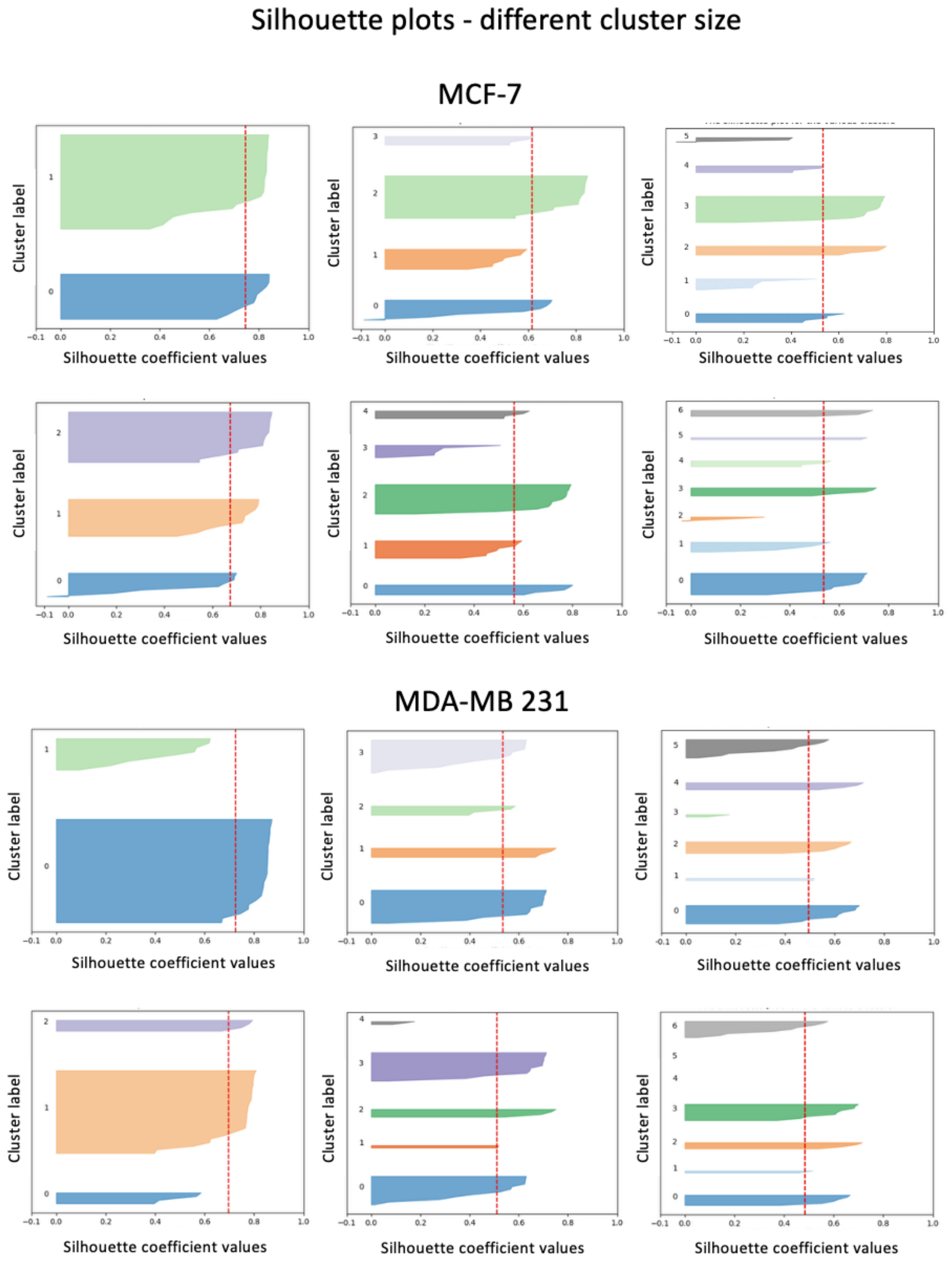

Figure SI.1. Silhouette plots of retrieved cellular phenotypes (from all microenvironment) when different cluster sizes (k=2 to k=7) are chosen for MCF-7 cells (top) and MDA-MB231 cells (bottom)

SI.2 Feature importance of cell phenotypes in all microenvironments

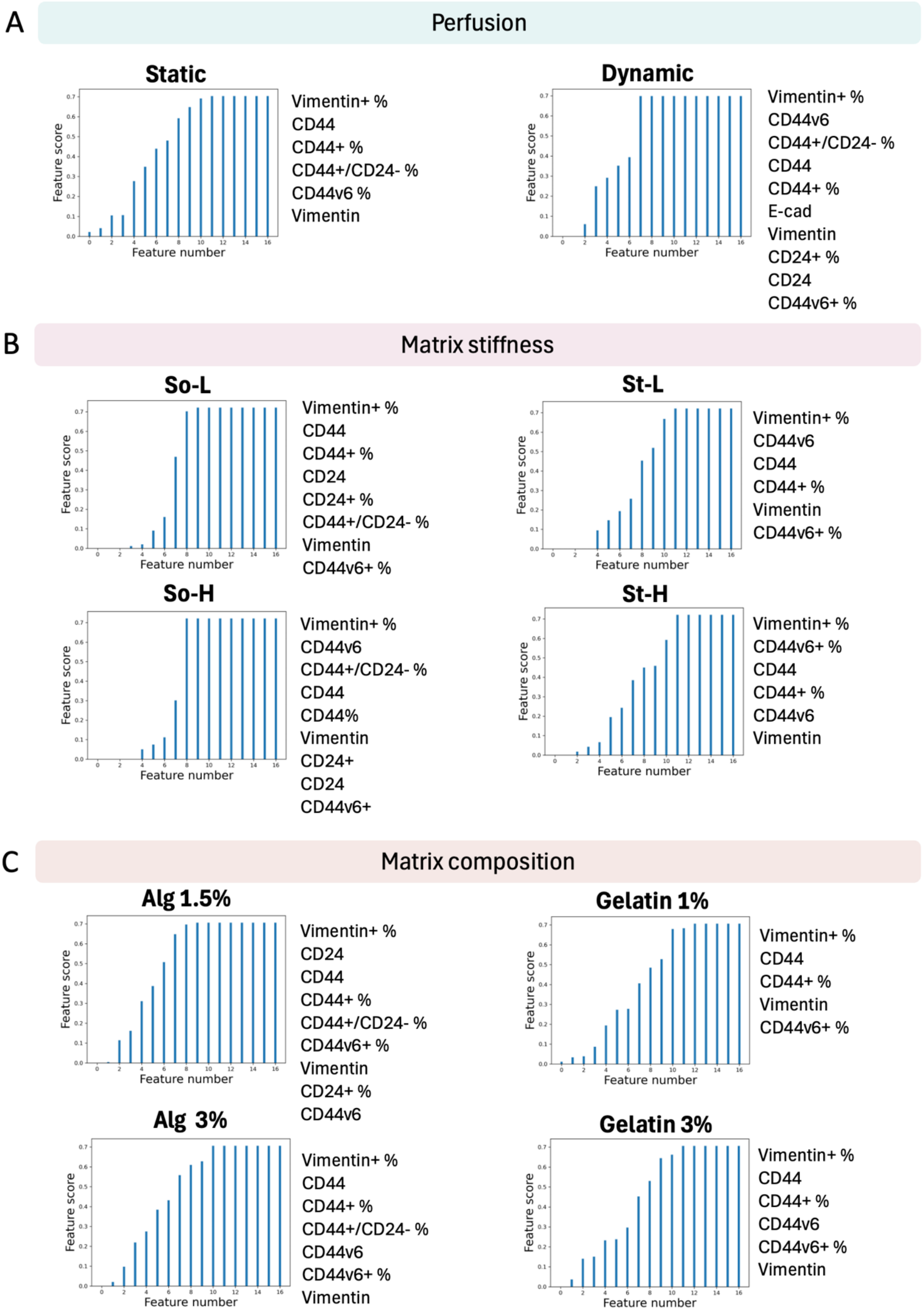

Figure SI.2. Cellular phenotypes/ features ranked from lowest to highest based on their feature importance in distinguishing MCF-7 vs MDA-MB231 in varying **A)** Perfusion status, **B)** Matrix stiffness and **C)** Matrix composition. The highest ranking features with equal importance score are listed on the right of each graph.

SI.3 Ranked features

Table SI.1. List of ranked features/markers in each microenvironment, ranked lowest to highest based on their importance in distinguishing invasive features

**pH 6.5**	**pH 7.4**	**Static**	**Dynamic**	**So-L 1.8**	**So-H 2.4**	**St-L 6.1**	**St-H 10.1**	**Alg 1.5%**	**Alg 3%**	**Gel 1%**	**Gel 3%**
ALDH	ALDH	Prol Day1	E-cad+ (%)	Prol Day1	ALDH	Prol Day1	Prol Day4	ALDH	ALDH	ALDH	ALDH
Prol Day1	Prol Day4	ALDH	ALDH	Prol Day7	Prol Day7	ALDH	ALDH	Prol Day1	alginate	Prol Day1	Prol Day4
Prol Day4	Prol Day14	Prol Day4	Prol Day14	Prol Day4	Prol Day14	Prol Day14	Prol Day7	E-cad+ (%)	Prol Day4	Prol Day14	Prol Day1
Prol Day7	E-cad+ (%)	Prol Day7	Prol Day1	Prol Day14	Prol Day4	CD24+ (%)	Prol Day14	Prol Day4	CD24+ (%)	Prol Day4	Prol Day7
CD44v6	Prol Day1	Prol Day14	Prol Day4	ALDH	Prol Day1	Prol Day7	Prol Day1	Prol Day14	Prol Day7	Prol Day7	Prol Day14
Prol Day14	Prol Day7	CD24+ (%)	Prol Day7	E-cad+ (%)	E-cad+ (%)	E-cad	CD24+ (%)	Prol Day7	Prol Day14	E-cad+ (%)	E-cad+ (%)
Vim	CD24+ (%)	E-cad	CD44v6+ (%)	E-cad	E-cad	Prol Day4	E-cad	E-cad	E-cad	CD24+ (%)	CD24+ (%)
CD24	E-cad	E-cad+ (%)	CD24	CD44v6	CD44v6+ (%)	CD44+ / CD24- (%)	E-cad+ (%)	CD44v6	E-cad+ (%)	E-cad	E-cad
E-cad	CD24	CD24	CD24+ (%)	CD24	CD24	E-cad+ (%)	CD44+ / CD24- (%)	CD24+ (%)	CD24	CD44+ / CD24- (%)	CD44+ / CD24- (%)
E-cad+ (%)	CD44v6	CD44v6	Vim	CD24+ (%)	CD24+ (%)	CD24	CD24	Vim	Vim	CD24	CD24
CD44v6+ (%)	CD44v6+ (%)	Vim	E-cad	Vim	Vim	CD44v6+ (%)	Vim	CD44v6+ (%)	CD44v6+ (%)	CD44v6	Vim
CD44+ /	Vim	CD44v6+ (%)	CD44+ (%)	CD44+ /	CD44+ (%)	Vim	CD44v6	CD44+ /	CD44v6	CD44v6+ (%)	CD44v6+ (%)

CD24- (%)				CD24- (%)				CD24 - (%)			
CD44 + (%)	CD44 + (%)	CD44 + / CD24- (%)	CD44	CD44 + (%)	CD4 4	CD44 + (%)	CD44 + (%)	CD44 + (%)	CD44 + / CD24- (%)	Vim	CD44 v6
CD44	CD44 + / CD24- (%)	CD44 + (%)	CD44 + / CD24- (%)	CD44	CD4 4+/C D24- (%)	CD44	CD44	CD44	CD44 + (%)	CD44 + (%)	CD44 + (%)
CD24 + (%)	CD44	CD44	CD44 v6	CD44 v6 + (%)	CD4 4v6	CD44 v6	CD44 v6	CD24	CD44	CD44	CD44
Vim + (%)	Vim + (%)	Vim + (%)	Vim + (%)	Vim + (%)	Vim + (%)	Vim + (%)	Vim + (%)	Vim + (%)	Vim + (%)	Vim + (%)	Vim + (%)
